# Early HELLP Syndrome or Catastrophic Antiphospholipid Syndrome? A Diagnostic Dilemma

**DOI:** 10.7759/cureus.38244

**Published:** 2023-04-28

**Authors:** Suhwoo Bae, Lizelle Comfort, Jason Ng, Kumar Sarkar, Sarah Pachtman

**Affiliations:** 1 Internal Medicine, Donald and Barbara Zucker School of Medicine at Hofstra/Northwell, Manhasset, USA; 2 Maternal Fetal Medicine, Donald and Barbara Zucker School of Medicine at Hofstra/Northwell, Manhasset, USA; 3 Cardiology, Donald and Barbara Zucker School of Medicine at Hofstra/Northwell, Manhasset, USA

**Keywords:** thrombocytopenia in pregnancy, medical disorders in pregnancy, catastrophic antiphospholipid antibody syndrome (caps), antiphospholipid antibody, hellp

## Abstract

Hypertensive disorders of pregnancy typically occur in the third trimester, with earlier presentations associated with underlying disorders such as antiphospholipid syndrome (APLS). We describe a case of a young primigravida presenting at 15 weeks 6 days gestation with epigastric pain, vomiting, new-onset severe-range hypertension, and subsequent development of anemia, thrombocytopenia, and transaminitis. Antiphospholipid antibodies (aPL) were triple-positive and imaging was negative for thrombosis. She was treated with aspirin, therapeutic anticoagulation, and ultimately dilatation and evacuation with initial postoperative improvement. Her symptoms returned postoperative day 3 and resolved following re-initiation of therapeutic anticoagulation. The differential diagnosis for hypertensive disorders of pregnancy is wide, particularly in second-trimester gestation, and includes catastrophic antiphospholipid syndrome (CAPS), lupus flare, microangiopathic anemias, and acute fatty liver of pregnancy. This case was an atypical presentation not clearly explained by any of the above diagnoses and required a multidisciplinary approach. Obstetric patients with high-risk aPL must be meticulously investigated with a broad differential to guide diagnosis and treatment.

## Introduction

Hemolysis, elevated liver enzymes, low platelets (HELLP) syndrome is a hypertensive disorder of pregnancy typically characterized by its named lab abnormalities [1]. HELLP syndrome occurs in about 0.5-0.9% of pregnancies and typically occurs in the third trimester, with peak frequency between 27-37 weeks gestation and only 10% occurring before 27 weeks [2]. HELLP syndrome can be diagnostically challenging given its similarities to other microangiopathic pathologies. Disorders that may present similarly to HELLP syndrome include acute fatty liver of pregnancy, thrombotic microangiopathies, and exacerbation of systemic lupus erythematosus [3]. Antiphospholipid antibodies (aPL) are a heterogeneous group of autoantibodies recognizing epitopes expressed by negatively charged phospholipids, proteins, or a protein-phospholipid complex associated with several clinical problems including arterial and venous thrombosis [4]. Catastrophic antiphospholipid syndrome (CAPS) is defined as multiple thromboses resulting in multi-organ failure and may be triggered by pregnancy [4]. HELLP syndrome can also imitate CAPS, but both disorders may manifest simultaneously with CAPS more likely to present de novo in pregnancy (48.2%) compared to non-pregnancy (26.3%), and also presenting with HELLP syndrome in 53% of pregnant women [4,5]. Here we present a case of a 21-year-old female G1P0 found to have triple-positive antiphospholipid antibodies that presented with symptoms concerning for early HELLP syndrome at 16 weeks and 0 days gestation initially treated with therapeutic enoxaparin as well as dilatation and evacuation, but with return of symptoms postoperatively with resolution by reinitiating therapeutic enoxaparin.

## Case presentation

A 21-year-old primigravida female presented at 15 weeks 6 days gestation dated by first-trimester sonogram with severe upper abdominal pain, nausea, and gastric vomiting, with no diarrhea or sick contacts. She was previously seen at an outside emergency room for similar symptoms, diagnosed with gastritis, and discharged. She reported no prior medical or surgical history. Family history was significant for a recent fatal thromboembolic event in the patient’s mother and two sisters with recurrent pregnancy loss.

Blood pressure on presentation was 171/104, with physical exam notable for epigastric abdominal tenderness on palpation and negative Murphy’s sign. Sonogram demonstrated a live intrauterine pregnancy measuring 14 weeks 5 days. Labs were remarkable for mild thrombocytopenia, mild leukocytosis, elevated fibrinogen, and prolonged aPTT/INR. Hemoglobin, LDH, haptoglobin, transaminases, and bilirubin were normal on presentation. COVID testing was negative. The patient was found to be antiphospholipid antibody triple-positive with positive lupus anticoagulant profile (positive Diluted Russell's Viper Venom Time, positive Silica Clotting Time but normal Dilute Thrombin Time), with high anticardiolipin (IgM 80.2 MPL, IgG 30.6 GPL) and anti-beta 2 glycoprotein I (IgG 52.8 SGU, IgM 141.7 SMU) (Table 1).

**Table 1:** Laboratory findings on admission WBC = white blood cells; HGB = haemoglobin; HCT = haematocrit; BUN = blood urea nitrogen; ALT: alanine transaminase; AST: aspartate aminotransferase; LDH: lactate dehydrogenase; aPTT: activated partial thromboplastin time; INR: international normalised ratio; DRVVT:Diluted Russell's Viper Venom Time; S/C: screen ratio/confirm ratio; IgG: immunoglobin G; IgM: immunoglobulin M

Test	Result	Reference Range
WBC	10.92	3.80-10.50 K/uL
HGB	12.2	11.5-15.5 g/dL
HCT	34.3	34.5-45.0%
Platelets	131	150-400 K/uL
BUN	4	7-23 mg/dL
Creatinine	0.46	0.50-1.30 mg/dL
Sodium	136	135-145 mmol/L
Potassium	3.8	3.5-5.3 mmol/L
Chloride	102	98-107 mmol/L
Bicarbonate	20	22-31 mmol/L
Calcium	9.7	8.4-10.5 mg/dL
ALT	23	4-33 U/L
AST	26	4-32 U/L
Total bilirubin	0.5	0.2-1.2 mg/dL
LDH	213	135-225 U/L
Fibrinogen	696	330-520 mg/dL
aPTT	88.4	27-36.3 sec
Prothrombin Time	13.6	13.4 sec
INR	1.17	0.88-1.16 ratio
Haptoglobin	139	34-200 mg/dL
Dilute Thrombin Time	18.6	16.0-26.0 sec
DRVVT Time	118.7	27.0-42.1 sec
Silica Clotting Time S/C Ratio	3.71	0.89-1.35 ratio
Anticardiolipin IgG, Serum	30.6	0.0-12.5 GPL
Anticardiolipin IgM, Serum	80.2	0.0-12.5 MPL
Beta 2 Glycoprotein 1 IgG Antibody	52.8	≤20.0 SGU
Beta 2 Glycoprotein 1 IgM Antibody	141.7	≤20.0 SMU

Hematology and rheumatology were consulted. Following immediate-acting antihypertensives, she was started on long-acting nifedipine, aspirin, and therapeutic enoxaparin. CT angiography of the abdomen and pelvis ruled out thrombotic mesenteric ischemia.

**Figure 1:**
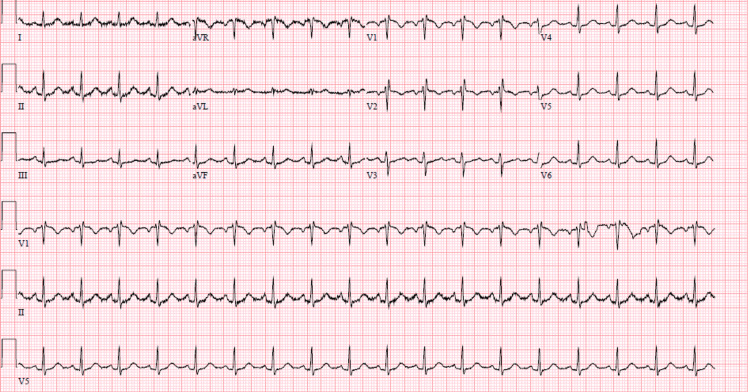
Electrocardiogram showing sinus tachycardia with incomplete right bundle branch block

Overnight, the patient developed pleuritic chest pain, and an electrocardiogram was obtained showing sinus tachycardia, no ST-segment changes but incomplete right bundle branch block, and T wave inversions in V1-V2 (Figure 1).

Repeat labs showed worsening thrombocytopenia (platelet nadir of 46 K/uL), normal hemoglobin but elevated lactate dehydrogenase (maximum 535) with no schistocytes on peripheral smear, mild hyperbilirubinemia (maximum of 2.4 mg/dL), and new transaminitis (maximum AST/ALT 205/156). Troponin T, high sensitivity assay was normal but pro-B-type natriuretic peptide was elevated at 921 pg/mL (reference range ≤299 pg/mL). An urgent transthoracic echocardiogram was performed with no evidence of right ventricular strain and normal left ventricular systolic function (Video 1).

**Video 1:** Apical 4-chamber view of transthoracic echo showing no evidence of right ventricular enlargement or strain

**Figure 2:**
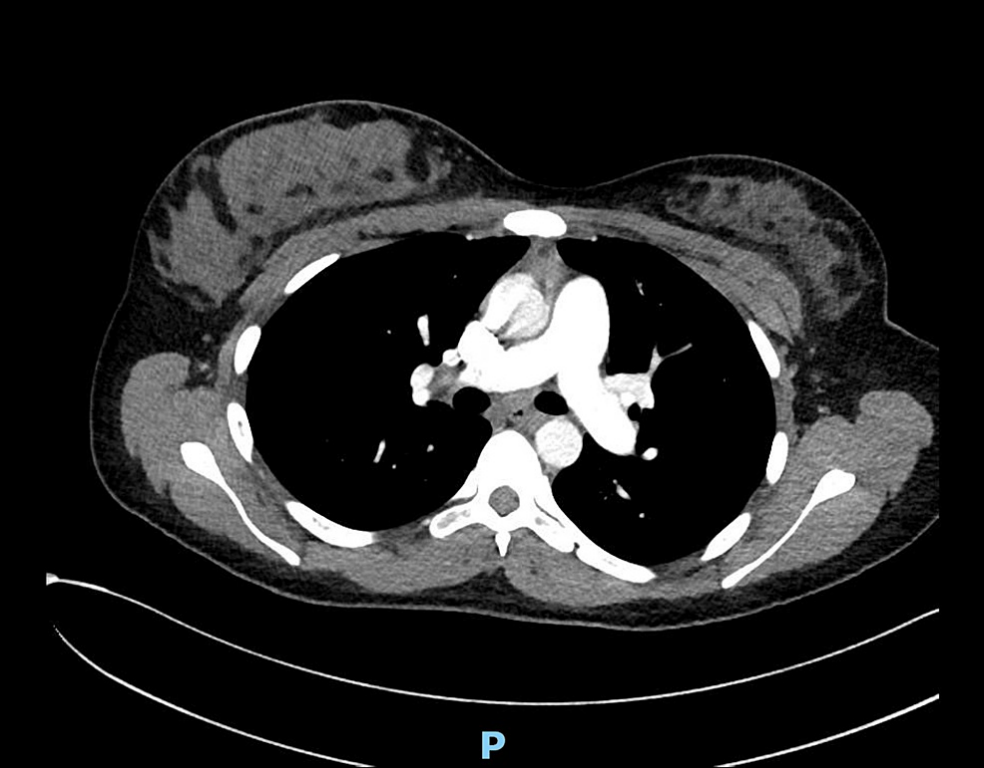
Computed tomography angiography showing no evidence of pulmonary embolism

A lower extremity doppler and CT chest angiogram respectively ruled out deep venous thrombosis and pulmonary embolism (Figure 2).

Following extensive discussion among a multidisciplinary team, in the absence of thrombosis, the patient was diagnosed with HELLP syndrome, and pregnancy termination via dilatation and evacuation was recommended. Given the uncertainty of the diagnosis, further treatment of CAPS with IVIG and steroid treatment was held off as there was no definitive proof on imaging of thrombi. Magnesium sulfate was administered for seizure prophylaxis and mifepristone/misoprostol were given for pre-operative cervical ripening. The patient underwent uncomplicated dilatation and evacuation with an estimated blood loss of 75 mL and was transfused one unit platelets intra-op given the rapidly worsening thrombocytopenia. She was transferred to the medical intensive care unit for close monitoring. One day post-op, hypertension resolved, and nifedipine was discontinued. Labs showed the resolution of thrombocytopenia and transaminitis. The patient developed postoperative anemia (nadir of 8.9 g/dL) that resolved without transfusion by postoperative day 3. Aspirin was continued while enoxaparin was decreased to prophylactic dosing.

On postoperative day 3 the patient had a return of epigastric pain, pleuritic chest pain, hypertension, thrombocytopenia, anemia, and transaminitis. Repeat CT angiogram of the abdomen/pelvis and abdominal ultrasound with Doppler studies demonstrated no visible thrombosis. After extensive multidisciplinary discussion, the patient was restarted on nifedipine and therapeutic enoxaparin on postoperative day 5. She again improved clinically, with the resolution of her symptoms and lab abnormalities, and further treatment for CAPS was once again held off. Other work-up performed to rule out other etiologies, such as viral/autoimmune hepatitis, were unremarkable. She was discharged on postoperative day 7 with close follow-up with the above consulting services. Fetal and placental pathology were remarkable for a fetus measuring approximately 14 weeks and accelerated villous maturation and parenchymal infarct. She was discharged on therapeutic enoxaparin and nifedipine and was transitioned to warfarin and aspirin on a follow-up visit with rheumatology. Repeat aPL drawn 12 weeks after initial presentation remained triple-positive.

## Discussion

We present a previously healthy patient with triple aPL positivity with a clinical diagnosis of HELLP syndrome who presented early in the second trimester, initially improved following pregnancy termination with clinical resolution following administration of therapeutic enoxaparin. Family history of venous thromboembolism and recurrent pregnancy loss in the setting of initially suspected CAPS led to prompt diagnostic testing for aPL [6].

There is a reported association between APLS and early-onset HELLP syndrome. A retrospective analysis of HELLP syndrome cases diagnosed below 26 weeks gestation reported only 6 total cases, all of whom had APLS [7]. Another retrospective case series at a large obstetric tertiary care center found that about half of early-onset second-trimester HELLP cases were associated with APLS, with the earliest reported case occurring at 18 weeks [8,9]. Our case is an example of early second-trimester HELLP syndrome with concurrent APLS/suspected CAPS with postoperative improvement following therapeutic anticoagulation.

Disorders such as preeclampsia and APLS have associated abnormalities in placental formation, with areas of infarction/necrosis, impaired spiral artery remodeling, and inflammation [10,11]. The process of abnormal vascular remodeling has been pathologically identified as early as the first trimester and precedes clinical manifestation by weeks to months [12,13].

CAPS is a condition with similar clinical manifestations of HELLP syndrome and should remain on the differential diagnosis for atypical presentations of the preeclampsia spectrum. A preliminary classification criteria for CAPS was proposed during the 10th International Congress on aPL in 2002 to facilitate the diagnosis of this potentially life-threatening condition defined by 4 criteria (Table 2) [14].

**Table 2:** Preliminary criteria for the classification of catastrophic antiphospholipid syndrome (CAPS) as proposed by the 10th International Congress on aPL

Criteria:
1. Evidence of involvement of three or more organs/tissues
2. Development of manifestations simultaneously or in less than one week
3. Histological evidence of intravascular thrombosis
4. Presence of antiphospholipid antibodies on two occasions six weeks apart
Definite CAPS:
1. All four criteria are met
Probable CAPS:
1. All four criteria except for only two organs/tissues
2. All four criteria except for confirmation of antiphospholipid antibodies due to patient death
3. Criteria 1, 2, 4
4. Criteria 1, 3, 4 with development of a third event between one week and one month after presentation, despite anticoagulation

The diagnosis of CAPS may be difficult and impractical. Suspicion with prompt treatment should be initiated if clinical concern increases for multisystem microangiopathic involvement in a patient with a known history of APLS, aPL positivity, or associated autoimmune disease [15]. A review of the “CAPS Registry” identified 15 cases of CAPS developing during pregnancy and postpartum, about half of whom concurrently developed HELLP syndrome, further complicating an already difficult diagnosis [16]. Current data support anticoagulation as first-line therapy for CAPS. Besides inhibition of clotting and degradation of existing clots, anticoagulation with heparin may have anti-inflammatory properties [5]. In general, the treatment of CAPS is directed at thrombotic events as well as suppression of the cytokine cascade. Besides anticoagulation, other therapies targeting the cytokine cascade have been reviewed by the Task Force on CAPS with a Grade B recommendation for the use of triple therapy (anticoagulation, glucocorticoids, plasma exchange, and/or IVIG) with a triple therapy strategy improving mortality compared to other strategies that did not use plasma exchange, IVIG or both [17]. Given the uncertainty of diagnosis as well as clinical improvement with therapeutic enoxaparin, our patient was not treated with triple therapy. Biologic drugs such as rituximab and eculizumab may also have a role in refractory APLS or CAPS, but there is limited data and these are currently recommended as a second-line therapy in patients refractory to standard triple therapy [17]. Anti-malarial drugs, specifically hydroxychloroquine, have been explored as an adjunctive therapy for thrombosis in APLS patients, but there has not been any high-quality data to recommend it as a first-line treatment. However, a recent randomized prospective study shows promise with a reduction in thrombotic events and no statistically significant differences in safety outcomes [18].

Data is limited on the management of obstetric APLS patients. In patients with a history of thrombotic APLS, combination treatment with low-dose aspirin and heparin at therapeutic dosages during pregnancy is recommended based on observational studies [15,19]. Definitive treatment of HELLP syndrome is delivery of the placenta. However, the optimal diagnostic approach and management of patients with concurrent HELLP syndrome and CAPS is unclear. The overlap between the presentation of CAPS and HELLP illustrates a difficult diagnostic dilemma requiring a multidisciplinary approach to ensure optimal outcomes.

## Conclusions

This case illustrates the association of APLS and HELLP syndrome and the diagnostic challenge in distinguishing between HELLP syndrome and CAPS. While high-risk aPLs are associated with the development of CAPS or HELLP syndrome during pregnancy, it is possible for a patient to present with a clinical picture composed of both disorders. Given the complexity and rarity of these disorders, a multidisciplinary approach is warranted to optimize patient outcomes. Obstetric patients with high-risk aPL must be meticulously investigated with a broad differential as HELLP syndrome in the early second trimester is exceptionally rare with other etiologies such as CAPS requiring alternative approaches to management.
